# RedTell: an AI tool for interpretable analysis of red blood cell morphology

**DOI:** 10.3389/fphys.2023.1058720

**Published:** 2023-05-26

**Authors:** Ario Sadafi, Maria Bordukova, Asya Makhro, Nassir Navab, Anna Bogdanova, Carsten Marr

**Affiliations:** ^1^ Institute of AI for Health, Helmholtz Zentrum München—German Research Center for Environmental Health, Neuherberg, Germany; ^2^ Chair for Computer Aided Medical Procedures & Augmented Reality, Technical University of Munich, Garching, Germany; ^3^ Red Blood Cell Research Group, Institute of Veterinary Physiology, Vetsuisse Faculty and the Zurich Center for Integrative Human Physiology, University of Zurich, Zurich, Switzerland; ^4^ Computer Aided Medical Procedures, Johns Hopkins University, Baltimore, MD, United States

**Keywords:** microscopic image analysis, single red blood cells, morphological features extraction, vesicle detection, deep learning, interpretable machine learning, segmentation, classification

## Abstract

**Introduction:** Hematologists analyze microscopic images of red blood cells to study their morphology and functionality, detect disorders and search for drugs. However, accurate analysis of a large number of red blood cells needs automated computational approaches that rely on annotated datasets, expensive computational resources, and computer science expertise. We introduce RedTell, an AI tool for the interpretable analysis of red blood cell morphology comprising four single-cell modules: segmentation, feature extraction, assistance in data annotation, and classification.

**Methods:** Cell segmentation is performed by a trained Mask R-CNN working robustly on a wide range of datasets requiring no or minimum fine-tuning. Over 130 features that are regularly used in research are extracted for every detected red blood cell. If required, users can train task-specific, highly accurate decision tree-based classifiers to categorize cells, requiring a minimal number of annotations and providing interpretable feature importance.

**Results:** We demonstrate RedTell’s applicability and power in three case studies. In the first case study we analyze the difference of the extracted features between the cells coming from patients suffering from different diseases, in the second study we use RedTell to analyze the control samples and use the extracted features to classify cells into echinocytes, discocytes and stomatocytes and finally in the last use case we distinguish sickle cells in sickle cell disease patients.

**Discussion:** We believe that RedTell can accelerate and standardize red blood cell research and help gain new insights into mechanisms, diagnosis, and treatment of red blood cell associated disorders.

## Introduction

Hematologists analyze human red blood cells (RBCs) under the microscope to detect abnormalities in RBC morphology, and to study and diagnose disorders (Bouyer et al., 2011; Makhro et al., 2016; Fermo et al., 2017). This manual process is time-consuming, subjective and prone to variability and errors. Moreover, whenever a vast number of single cells needs to be analyzed, manual analysis is impossible, making automatic, high throughput image analysis an essential element in microscopy based RBC research.

Artificial Intelligence (AI) is emerging as one of the main technologies enabling automated processing of blood microscopy images (Deshpande et al., 2021). A typical workflow includes segmentation, feature extraction and classification based on extracted RBC features (Devi et al., 2018; Petrović et al., 2020; Wong et al., 2021). Segmentation is commonly performed through classical computer vision algorithms including operations such as thresholding, image filtering, morphological operators, etc. followed by watershed transforms (Sharif et al., 2012) and ellipse fitting (Naruenatthanaset et al., 2020) to separate single cells. Multi-purpose computational tools provide segmentation algorithms based on the mentioned methods and enable feature extraction of single cell morphology, with CellProfiler (Stirling et al., 2021) and ImageJ (Schneider et al., 2012) being the two most commonly used open-source softwares for microscopy image analysis. However, pipelines and macros require the optimization of many hyperparameters, which are specific for images and experimental setups, and thus do not generalize well to other datasets (Sinha et al., 2015). Alternatively, machine learning methods are employed. For instance, Savkare and Narote (2015) suggest segmenting cells with a k-mean clustering followed by cell separation using a watershed algorithm. In a more modern approach, Wong et al. (2021) use a U-Net (Ronneberger et al., 2015) model to segment RBCs followed by a support vector machine classifier (Suykens and Vandewalle, 1999) to separate overlapping cells, cell morphological features are extracted and used for a cell type classification task with a TabNet (Arik and Pfister, 2019) model. In one of our previous works, we suggest a fully convolutional Alexnet architecture to segment single RBCs using a sliding window approach to prevent overlapping segmentation (Sadafi et al., 2018). A more robust strategy (Dhieb et al., 2019) suggests a cell counting method based on a Mask R-CNN (He et al., 2017), an architecture able to distinguish between single cells, and thus requiring no further post-processing to separate overlapping detections.

As for the classification of the single cells, rule-based approaches, k-nearest neighbors, support vector machines or other classical machine learning methods have been employed (Akrimi et al., 2014; Piety et al., 2015). Specifically, tree-based classifiers such as random forest or gradient boosting were shown to be superior when distinguishing between normal, sickle and other abnormally shaped RBCs based on the previously extracted features (Petrović et al., 2020). Also deep learning based methods were considered for RBC classification. For instance (Alzubaidi et al., 2020; Parab and Mehendale, 2021), and (Savkare and Narote, 2015; Naruenatthanaset et al., 2020) use deep convolutional neural networks for this purpose (Sadafi et al., 2019; Song et al., 2021). apply Fast R-CNN and Faster R-CNN models to differentiate between 14 and 7 RBC subtypes, respectively. The most recent work suggests a pipeline based on deep ensemble learning for RBC morphology classification (Routt et al., 2023). Such methods result in a higher classification accuracy in multi-class settings. However, they take images as input and process them to obtain a useful representation of features, which are completely model generated and are not interpretable as opposed to extraction of hand-crafted features. Moreover, they require a large amount of annotated data to achieve a proper generalization power. As a rule, most work on RBC classification mainly focuses on fitting a classifier for a specific task and specific dataset. Extension of deep classification models to a new task or dataset requires programming skills and computer science expertise to gain a deep understanding of algorithms and adjust them properly. Additionally, their application on a new task requires understanding and running source code, which is not always given (Wagner et al., 2022).

This paper introduces RedTell, an AI tool for interpretable analysis of RBC morphology. RedTell enables single-cell segmentation, extraction of cell morphology features, and RBC classification without any prior knowledge and extensive user interaction. RedTell is a fully automated tool expecting expert input only for cell annotations. It can assist a wide range of research questions by 1) providing a robust RBC segmentation model with the possibility of adaptation to new datasets with or without fine-tuning, 2) extracting a wide range of RBC morphological features as described in the literature (Veluchamy, 2012; Devi et al., 2018) and 3) enabling task- and dataset-specific explainable classification algorithms with a low amount of annotated input images. The results of every step of the RedTell pipeline can be directly used for the downstream analysis. RBC counting, comparison of cell type and feature distribution in different experiments or sample groups, or diagnosis of diseases with irregular cell morphology (such as sickle cell disease) are just some of the possible scenarios where RedTell can be applied. We showcase the abilities of RedTell in three different case studies. First, we show that extracted features have different distributions for healthy and anemia patients. Next, we use RedTell to distinguish cells in the stomatocyte-discotye-echinocyte (SDE) sequence in control samples. Finally, we use RedTell to classify sickle cells in samples of sickle cell disease patients.

The novelty of RedTell includes feature extraction from images in fluorescent (in our case Fluo-4 was used as a fluorescent dye to detect Ca2+) channel, vesicle detection in the Fluo-4 channel, and the increased interpretability: we explicitly provide segmentation and classification results overlaid on the original images, meaningful key characteristics of RBCs widely used by hematologists and feature importance to explain predictions of classification algorithms.

## Materials and methods

RedTell is an AI tool developed to facilitate the analysis of microscopic images of human RBCs. It consists of four steps:

1. Accurate cell segmentation
2. Extraction of hand-crafted morphological features from brightfield and fluorescence (Ca2+-dependent Fluo-4 signal as an example) channels including vesicle detection and counting
3. Assistance in data annotation
4. Single RBC classification

**FIGURE 1:**
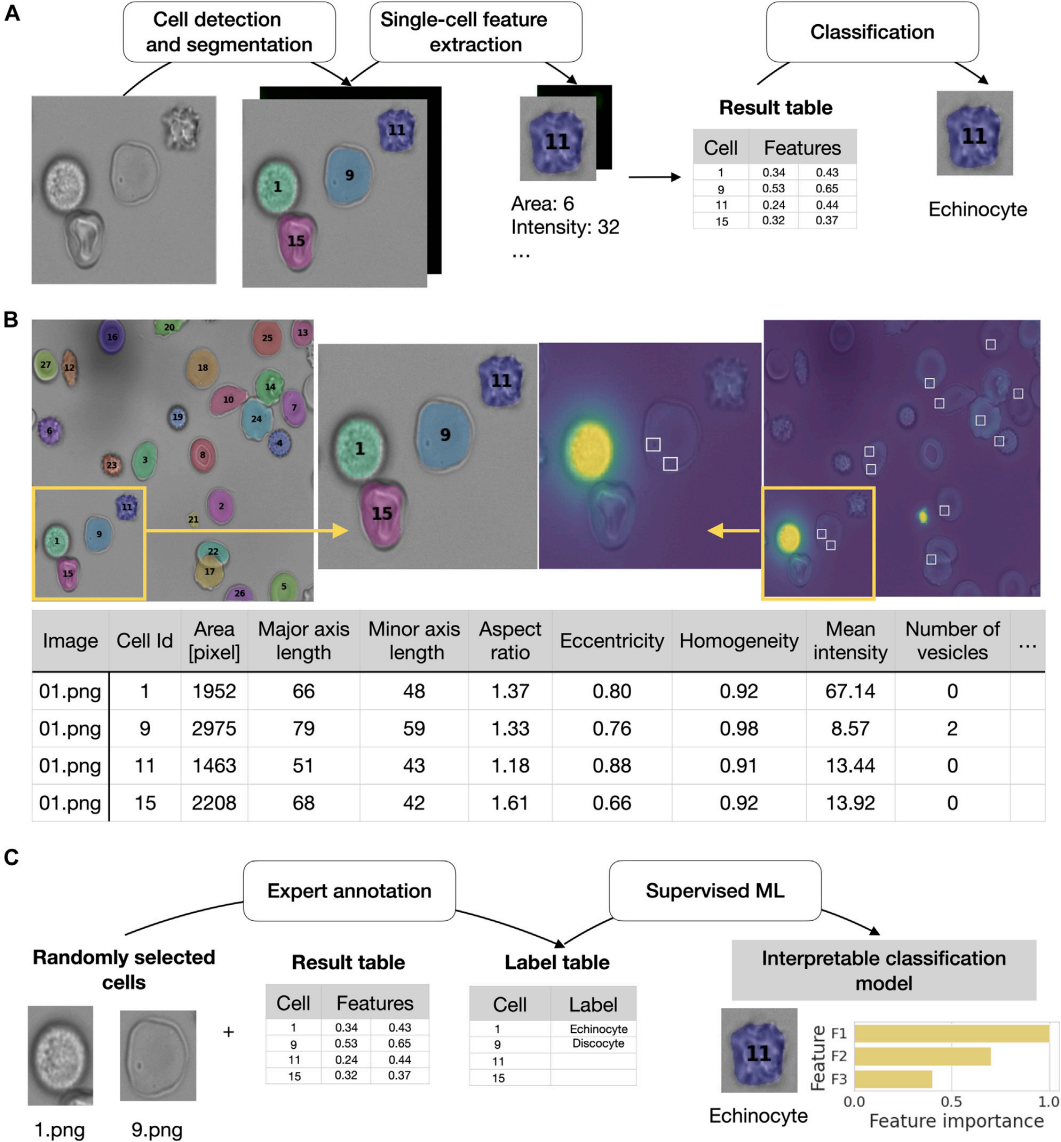
RedTell facilitates single red blood cell profiling by extracting interpretable features and enabling accurate cell classification. **(A)** Overview of the RedTell functional pipeline: Single red blood cells in microscopy images are segmented, 135 morphological features are extracted, and cells are classified into different cell types. **(B)** RedTell provides a table with extracted features, brightfield images overlayed with segmentation results, and fluorescent images with detected vesicles highlighted white boxes. **(C)** RedTell supports annotation of single-cells for automated supervised machine learning based on interpretable features.

Every step can be executed by a simple command in the terminal. RedTell is a software package implemented in Python and is easily accessible through the command line under any operating system. Code and extensive documentation as well as CoMMiTMenT and MemSID datasets used in the manuscript are provided under https://github.com/marrlab/redtell. The software is distributed under MIT license without any restrictions for academic and non-academic use. An overview of RedTell’s functional pipeline is given in Figure 1A. In the following we describe each step in detail.

### Datasets

#### CoMMiTMenT and MemSID datasets

We use CoMMiTMenT dataset to develop the segmentation solution in RedTell as well as consider it to evaluate feature extraction and classification functionality of the tool. The dataset consists of 3 patients with Thalassemia, 9 patients with sickle cell disease, 8 patients with hereditary xerocytosis caused by mutation in PIEZO1 channel, and 13 patients with hereditary spherocytosis. 26 individuals are in a healthy control group.

An Axiocam mounted on a Axiovert 200 m Zeiss microscope with a ×100 objective was used to obtain the images. No staining or preprocessing is performed.

The CoMMiTMenT study was funded by the European Seventh Framework Program under grant agreement number 602121 (CoMMiTMenT) and from the European Union’s FP7 Programme. The study protocols were approved by the Medical Ethical Research Board of the University Medical Center Utrecht, the Netherlands, under reference code 15/426 M and by the Ethical Committee of Clinical Investigations of Hospital Clinic, Spain (IDIBAPS) under reference code 2013/8436. Additional blood samples of patients with sickle cell disease were obtained from the participants of the MemSID clinical trial performed at the University hospital Zurich (#NCT02615847 at https://clinicaltrials.gov/). The trial protocol was approved by the Ethics committee of Canton Zurich (KEK-ZH 2015-0297).

### External datasets

We also consider three publically available datasets of blood smear microscopic images: ThalassemiaPBS (Tyas et al., 2022) and MP-IDB (Loddo et al., 2019). Chula-RBC-12-Dataset (Naruenatthanaset et al., 2020) on ThalassemiaPBS consists of 80 images obtained from four thalassemia patients (20 images for each patient), and covers RBCs of various morphology. The raw images have no annotations. Cropped single-cell images are assigned in a separate dataset to one of 9 morphological subtypes. MP-IDB dataset consists of 229 images of patients affected by four different kinds of malaria parasite. Each image contains RBCs with at least 1 cell hosting a parasite. Only such cells are annotated with a segmentation mask and parasite’s kind and life-cycle stage. Chula-RBC-12-Dataset covers 706 images of RBC (no further details about patient pool are given). It includes labels for every RBC in 12 different morphological subtypes in form of point annotations, no segmentation masks are provided. Due to the limited availability of relevant annotations, we use the described datasets to qualitatively assess the generalization ability of our segmentation model.

### Cell segmentation

Accurate cell segmentation is the first essential step for studying morphological features of red blood cells. Single-cell segmentation masks provide exact cell localizations in the image and allow computation of precise cell features. We approach the segmentation task by using a Mask R-CNN model (He et al., 2017), an artificial neural network that extends R-CNN based models (Girshick et al., 2014; Girshick, 2015; Ren et al., 2015) consisting of two stages: 1) a region proposal network to propose and evaluate various locations of objects in the image, and 2) a three head network analyzing objects one by one for object classification and bounding box location refinement. Such architectures have been effective in various image segmentation scenarios (Chiao et al., 2019; Huang et al., 2019) and applied on red blood cell microscopy images with a large diversity of cellular morphologies (Dhieb et al., 2019; Sadafi et al., 2019; Sadafi et al., 2020; Loh et al., 2021). Mask R-CNN advances its forerunners by preserving spatial information of regions corresponding to the objects in the images and by having an additional, fully convolutional head for object segmentation.

RedTell provides a ready-to-use Mask R-CNN model trained on the brightfield images of the control samples from the CoMMiTMenT dataset annotated on the single-cell level as introduced by (Sadafi et al., 2020) as an accurate and robust segmentation solution. In the dataset, every image contains on average only 44.2 cells. Furthermore, RedTell enables automated training of new Mask R-CNN segmentation models on any custom dataset with annotated images provided.

### Feature extraction

RedTell supports capturing of valuable biological insights in the analysis of RBCs by extracting interpretable hand-crafted features for every segmented cell. Every feature represents a meaningful characteristic of a cell describing its morphology. The features are extracted from two modalities: 1) brightfield microscopy and 2) fluorescence microscopy. Both image types are converted to grayscale for feature acquisition.

#### Feature extraction from brightfield channel

The feature set provided by the RedTell can be categorized into three different groups:

- *Shape features*, which describe cell morphology and include characteristics such as area, length of minor and major axes, aspect ratio and eccentricity. The shape features are widely used to distinguish between normal and irregular RBC for automated diagnosis of RBC disorders such as sickle cell disease or thalassemia (Chy and Anisur Rahaman, 2019; Das et al., 2020; Tyas et al., 2020).

- *Intensity-based statistical features*, which describe cell properties derived from intensity distribution of pixels corresponding to a cell such as mean intensity, standard deviation, skewness and kurtosis. Such features were shown to be useful to differentiate between healthy and sickle RBCs based on the intensities of color and grayscale images. (Akrimi et al., 2014; Tyas et al., 2020). Intensity features are particularly meaningful when extracted from the images of the fluorescence microscopy as they provide insights about cell behavior measured by the activation of the fluorescence as reviewed in (Coffman and Wu, 2012).

- *Texture features*, which reflect local distribution of pixel intensities and characterize homogeneity and texture of a cell. For every cell we extract the histogram, Gray Level Co-occurrence Matrix (GLCM) and Gray Level Dependence Matrix (GLDM), which represent a relationship between adjacent pixels and include features such as contrast and dissimilarity. We further extract the Gray Level Size Zone Matrix (GLSZM) and the Gray Level Run Length Matrix (GLRLM) to indicate basic structures within a cell such as the size of the largest region of the same pixel intensity. Texture features have been successfully exploited for the RBC classification in blood smear images (Veluchamy, 2012; Akrimi et al., 2014; Chy and Anisur Rahaman, 2019).

Shape features are directly obtained from segmentation masks of the cells, while intensity and texture features are calculated after co-localizing the single-cell masks in the normalized images of brightfield and fluorescent channels. One of the biggest advantages of calculating features based on single-cell masks is focusing only on pixel values that are corresponding to a cell rather than its surrounding artifacts and noise.

#### Feature extraction from fluorescence channel

Segmentation masks obtained from the brightfield channel provide the exact cell positions. However, the brightfield and fluorescent channel images are produced sequentially. As the microscope stage and the imaging chamber containing RBCs may move during the switch from one channel to the other or due to the tumbling of RBC membrane, slight displacement in the position of a given cell may be introduced which becomes visible why sets of brightfield and fluorescent images are overlaid. An approach to train a separate Mask R-CNN model for cell detection and segmentation in a fluorescent channel requires additional annotation of the cells in fluorescent images and introduces a non-trivial task of the assignment of detections between brightfield and fluorescent channels. Instead, we propose applying segmentation masks obtained from the brightfield channel directly to the fluorescent channel images. We approach a possible slight shift in cell position by excluding image background defined as pixels with zero intensity from the feature extraction. This approach can be justified by the fact that intensity value distribution is homogenous or symmetric with respect to the cell center, and covering only a part of the cell provides a reasonable estimate for the entire object. We further eliminate for feature extraction pixels corresponding to the intracellular artifacts such as vesicles, which we detect with an algorithm as described next.

#### Vesicle detection

We further detect and count vesicles, intracellular compartments of RBCs as first described by (Lew et al., 1985) in sickle cell patients. The vesicles are inside-out facing parts of the plasma membrane containing the plasma membrane Ca2+ pump (PMCA). The latter effectively sequesters Ca2+ into the vesicles preventing activation of Ca2+-dependent K+ (Gardos) channels. The measurements of the abundance, size and Ca2+ storage capacity of the vesicles in RBCs provide information on the Ca2+ leak through the membrane and the ability of the cells to avoid Ca2+ overload. These tasks are best achieved by imaging of the average levels of free Ca2+ ions in living RBCs as well as the distribution of Ca2+ ions within the cells (between the cytosol and the intracellular vesicles) performed using Fluo-4 AM fluorescent dye. This dye was proven to be the best for use in RBCs due to its high fluorescence intensity with a high degree of quenching of the fluorescent signal by hemoglobin being produced (Kaestner et al., 2006; Wang et al., 2022).

Single snapshots provide information on the current levels of Ca2+ in RBCs and their compartments. Functional tests, in which Ca2+ uptake via the Ca2+-permeable channels is stimulated mechanically or chemically, allow to monitor dynamics of Ca2+ movement across the membrane as time lapse image sequences are obtained for the same set of cells (Hänggi et al., 2014; Fermo et al., 2017; Makhro et al., 2017). These tests give indications on the abundance and/or activity of the Ca2+-permeable channels and PMCAs, that maintain the intracellular Ca2+ levels, that, in turn, are involved in regulation of RBC dehydration and deformability (Muravyov and Tikhomirova, 2012; Kaestner et al., 2020).

We can detect inside-out vesicles in the images of Fluo-4 channel as small regions with high intensity values inside the RBCs [e.g., (Hänggi et al., 2014)] as vesicles are filled with Ca2+ as it gets actively pumped into them by the plasma membrane Ca2+ ATPases against the gradient, and function as intracellular Ca2+ stores. Such intraerythrocytic compartmentalisation of Ca2+ is particularly prominent in RBCs of patients with sickle cell disease, where excessive membrane Ca2+ leak is compensated by Ca2+ sequestration into the internal stores preventing immediate terminal dehydration of the cells due to the opening of Ca2+-dependent K+ (Gardos) channels (Lew et al., 1985). Ca2+ overload in RBCs was shown to be a non-specific indicator of multiple hereditary hemolytic anemias and it is more pronounced in patients with severe disease manifestation (Hänggi et al., 2014; Hertz et al., 2017).

Therefore the number of vesicles found in every cell can be considered as a meaningful, clinically relevant feature. Moreover, vesicles having higher Ca2+ concentration can affect the value of intensity and texture features and should be ignored while calculating the features. We introduce an algorithm for vesicle detection and elimination by determining the local maxima of intensity values.

Let $I\in R^{X\times Y}$ be an image represented by pixel intensities. Then

$$U_{d}\left(x,y\right):=\left\{\left(\hat{x},\hat{y}\right)\in I\left|\left\|\left(\hat{x},\hat{y}\right)-\left(x,y\right)\right.\right|{|}_{1}<d\right\}$$

defines a set of pixel intensities in a ball with radius d>0 around the pixel $\left(x,y\right)\in I$ with respect to the $L^{1}$ distance and

$${\bar{U}}_{d}\left(x,y\right):=\frac{1}{\left|U_{d}\left(x,y\right)\right|}\underset{\left(\hat{x},\hat{y}\right)\in U_{d}\left(x,y\right)}{\sum }I\left(\hat{x},\hat{y}\right)$$

is the average intensity value in $U_{d}\left(x,y\right).$ The vesicle is then defined as a region $\left.U_{v}{\left(x\right.}^{⋆},y^{⋆}\right)$ around the local maximum ${\left(x\right.}^{⋆},y^{⋆})\in I$ , which satisfies

$${\bar{U}}_{v}\left(x^{⋆},y^{⋆}\right)-t\;>\frac{1}{\left|U_{c}\left(x^{⋆},y^{⋆}\right)\;\backslash U_{v}\left(x^{⋆},y^{⋆}\right)\right|}\underset{{\left(x,y\right)\in U}_{c}\left(x^{⋆},y^{⋆}\right)\;\backslash U_{v}\left(x^{⋆},y^{⋆}\right)}{\sum }I\left(x,y\right)$$

where v , c and t are predefined constants. v is an estimate of vesicle radius and the threshold t corresponds to the least assumed difference in mean intensity values inside a vesicle and a region around it. To determine the local maxima of the image we apply a median filter of size m×m and a maximum filter of size v . This ensures detection of at most one maximum within a single vesicle. We calculate local maxima on the whole original image of the fluorescent channel and map a detected vesicle to a specific cell if it is inside its segmentation mask. Thus, we limit the search for local maxima only on the image section belonging to the cell. The parameters m , d , t , v and c are dependent on the dataset and should be adjusted accordingly. For our dataset, we experimentally determined parameters which provide good vesicle detection results to be =3 , d=10 for local maximum determination, v=4 and c=8 for vesicle and neighborhood definition and 1 for intensity difference threshold between vesicle and neighborhood region.

We remove a vesicle by setting $I\left(x,y\right)=0$ for $\left.\left(x,y\right)\in U_{v}{\left(x\right.}^{⋆},y^{⋆}\right)$ . Pixels corresponding to the vesicles are artifacts within the cell image and treated similarly to the background pixels, they should not contribute to calculation of the intensity and texture features.

In total, we extract 135 features: 14 shape features from segmentation masks for the brightfield channel, 18 intensity and 42 texture features from each brightfield and fluorescent channels, and we further obtain the number of vesicles if the fluorescent channel is Ca2+ channel. We use python libraries skimage (van der Walt et al., 2014) and pyradiomics (van Griethuysen et al., 2017) for the extraction of shape features and intensity and texture features, respectively. Shape features are biologically most relevant, while intensity and texture features are primarily useful for classification. We list all extracted features in Table 1.

**TABLE 1:** RedTell extracts 135 interpretable morphological features of RBCs. The table provides a list of features for four feature groups. For every group we provide the source implementation of feature extraction in the *Extracted with* column. In particular, the detailed description of every feature and their computation formulas are provided in the documentation of source libraries. Column *Feature prefix* gives a prefix used for each feature group in the tables produced by RedTell after feature extraction.

Feature group	Extracted from	Feature prefix	Extracted with	Feature names
Shape features	Segmentation mask	shape	pyradiomics	Pixel Surface (Area)
				Perimeter
				Major Axis Length
				Minor Axis Length
				Maximum Diameter
				Perimeter Surface Ratio
				Sphericity
				Elongation
			skimage	Convex Area
				Bounding Box Area
				Extent
				Eccentricity
				Equivalent Diameter
				Solidity
Intensity-based statistical features	Brightfield and fluorescent channel	intensity	pyradiomics	Mean
				Variance
				Median
				Minimum
				Maximum
				Range
				10 Percentile
				90 Percentile
				Interquartile Range
				Mean Absolute Deviation
				Robust Mean Absolute Deviation
				Root Mean Squared
				Entropy
				Energy
				Total Energy
				Uniformity
				Skewness
				Kurtosis
Texture features	Brightfield and fluorescent channel	glcm	pyradiomics	Contrast
				Correlation
				Autocorrelation
				Difference Average
				Difference Entropy
				Difference Variance
				Inverse Difference (ID)
				Inverse Difference Moment (IDM)
				Informational Measure of Correlation 1 (IMC1)
				Informational Measure of Correlation 2 (IMC2)
				Inverse Variance
				Joint Average
				Joint Energy
				Joint Entropy
				Maximal Correlation Coefficient (MCC)
		gldm	pyradiomics	Dependence Entropy
				Dependence Non-Uniformity
				Dependence Variance
				Gray Level Non-Uniformity
				Gray Level Variance
				gldm_LowGrayLevelEmphasis
				gldm_HighGrayLevelEmphasis
		glszm	pyradiomics	High Gray Level Zone Emphasis
				Large Area Emphasis
				Large Area High Gray Level Emphasis
				Large Area Low Gray Level Emphasis
				Low Gray Level Zone Emphasis
				Size Zone Non-Uniformity
				Small Area Emphasis
				Small Area High GrayLevel Emphasis
				Small Area Low Gray Level Emphasis
		glrlm	pyradiomics	Long Run Emphasis
				Long Run High Gray Level Emphasis
				Long Run Low Gray Level Emphasis
				Low Gray Level Run Emphasis
				Run Entropy
				Run Length Non-Uniformity
				Run Percentage
				Run Variance
				Short Run Emphasis
				Short Run High Gray Level Emphasis
				Short Run Low Gray Level Emphasis
Vesicle features	Fluorescent channel	ca^2+^	RedTell original	Number of vesicles

### Cell classification

For many applications such as image-based disease diagnosis there is a need for classification of cells found in the images into specific cell types. Features extracted by RedTell are a valuable resource facilitating this challenging task. These features are successfully used in some relevant works to obtain methods to distinguish different cell types of interest (Tomari et al., 2014; Chy and Anisur Rahaman, 2019). In addition to feature extraction, RedTell also provides an automated training procedure for binary or multi-class classification algorithms without requiring extensive user interaction. Experts can train their task specific and dataset specific classifier by only annotating a small subset of the cells without the need for accessing sophisticated high performance computing resources. RedTell is designed to reduce the total number of annotations necessary for training of a classifier while achieving high classification accuracy (see Figures 7, 8).

#### Annotation

RedTell supports annotation procedure for generating ground truth necessary for training of classification algorithms. For this, it randomly selects N segmented cells with extracted features, saves single-cell images with unique identifiers and provides a label table containing a column of unique cell identifiers and a label column. (see Figure 1C). Looking at each single-cell image an expert determines the cell label and enters it into the table in the corresponding row. Random sampling of cells for annotation should provide various cells across different images of the dataset and groups, e.g., patient, diseases or experimental settings, to cover a wider range of cells of different morphology. It also ensures that class distribution of the annotated part of the dataset properly reflects class distribution of the entire dataset. Annotated cells with non-empty labels compose a training set for supervised classification The goal is to predict a cell type for non-annotated cells and include the results in the feature output table.

#### Classification algorithms

For classification, we suggest using tree-based algorithms decision tree, random forest and LightGBM as 1) they do not require a large amount of data to achieve a high classification accuracy and 2) are interpretable by providing feature importance when discriminating between cell classes in every decision node. Furthermore, random forest and LightGBM are ensemble methods aggregating predictions from multiple decision trees to increase accuracy, robustness, and prevent overfitting. All three algorithms are interpretable by design and allow measurement of feature importance in classification. Decision tree is built on optimizing an information criterion (entropy or GINI). The criterion determines which features to use for a node split when building a decision tree. Features with the highest information gain or decrease in impurity in case of entropy and GINI, respectively, are considered to have the largest impact on classification results. For the random forest classifier, for each feature its importance is calculated as average information gain or impurity decrease over all decision trees. In contrast, the LightGBM model does not use any information criterion to build the decision trees and is optimized with gradient boosting. Importance for each feature is given by the number of node splits utilizing the features calculated over all decision trees.

#### Automated machine learning

Development of a classifier is performed by means of Automated Machine Learning (AutoML) (He et al., 2019), which automatically determines the optimal hyperparameters for every classification algorithm included in the tool and provides the best model for a given dataset and classification problem. AutoML does not require from users any technical expertise, programming skills or deep understanding of algorithms. The RedTell AutoML module includes no.

- loading the dataset from the feature and label tables,
- generating stratified, image-based or random partitioning of the dataset for 5-fold cross validation,
- training of 75 classifiers (25 steps of Bayesian hyperparameter optimization for 3 classification models),
- selecting and saving the best performing model,
- generating model evaluation and model explanation artifacts, and
- applying the model to the unlabeled observations.

For hyperparameter tuning, we use a 5-fold cross validation with average balanced accuracy over 5 folds as an optimization objective. Optimization is performed via Bayesian optimization approach (Snoek et al., 2012). A partition of the annotated dataset into the 5 folds can be chosen between being completely random or in a stratified fashion taking the images into account such that observations coming from a single image are never included in both training and validation sets. Random partition is appropriate for smaller datasets where every image contains annotated cells. In contrast, image-based partition can assess generalization ability more realistic for a larger dataset where classification of cells on previously unseen images is expected. To approach a possible class imbalance in the dataset we use the inverse of class frequency as weights while optimizing the training objective. RedTell trains a decision tree classifier, a random forest, and LightBGM models with various sets of hyperparameters. After finding the optimal model with optimal hyperparameters, the model is fitted on the whole annotated set obtaining the final classification model. This classification model is then applied to the unlabeled observations for class predictions.

## Results

In this section, we first discuss and evaluate the proposed cell segmentation based on a Mask R-CNN and the vesicle detection, and then showcase the abilities of RedTell in different case studies. First, we show that extracted features are different for different RBC disorders. Next, we use RedTell to distinguish cells in the stomatocyte-discotye-echinocyte (SDE) sequence in control samples. Finally, we use RedTell to classify sickle cells in patient samples from the MemSID clinical trial.

### Red blood cell segmentation

**FIGURE 2:**
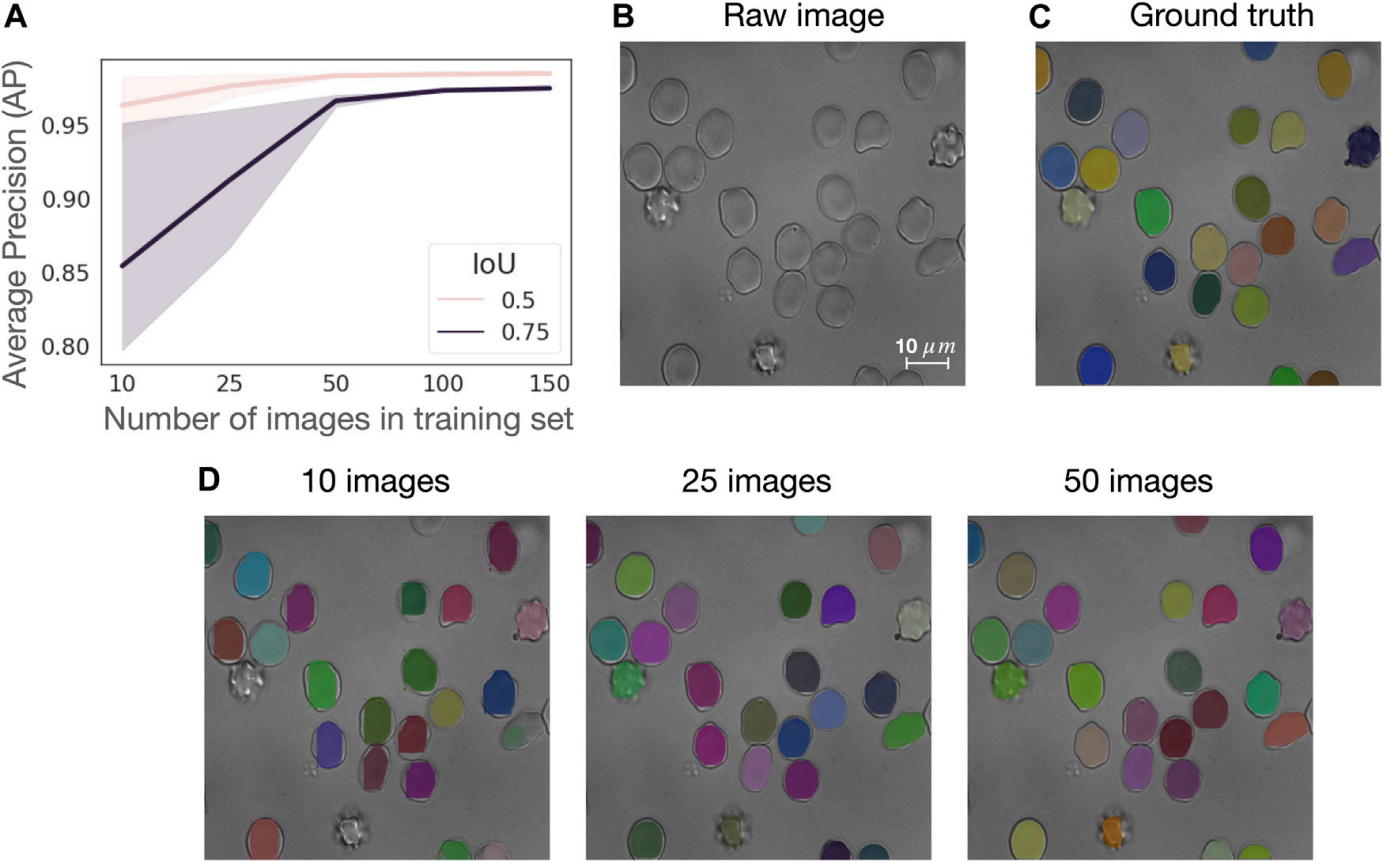
50 annotated images suffice to train a RBC Mask R-CNN segmentation model with a good performance. **(A)** Average precision (AP) curves for IoU of 0.5 and 0.75 calculated on three different train and test splits with a training set consistent of 10, 25, 50, 100, and 150 images, where each image contains about 44 single RBCs. **(B)** Sample image from the test set in one of the splits, **(C)** corresponding ground truth segmentation masks overlaid on raw image and **(D)** segmentation results achieved with models trained with datasets of 10, 25, and 50 images overlaid on raw image.

We assess the generalizability of the RBC Mask R-CNN segmentation model on a subset of control samples from the CoMMiTMenT dataset (see Materials and Methods) and investigate how the size of the training set affects model performance. Although RedTell provides a ready-to-use segmentation model, it also includes the option to train a new model for a custom dataset. Here, we are interested in estimating the number of annotated images necessary to obtain a good segmentation result. We thus consider 187 images from control samples containing 8222 annotated RBCs (73% discocytes, 21% echinocytes and 6% stomatocytes, see Figure 7A). We randomly split the data into 150 (80%) images for training and 37 (20%) for testing. From training, we further randomly sample 10, 25, 50, and 100 images to fit a Mask R-CNN model without adjusting model training hyperparameters for different sizes of the training set. We perform experiments on three different training and test splits to obtain confidence intervals for average precision (AP). As shown in Figure 2A, 50 annotated images suffice to achieve an average AP of 0.98 and 0.95 for IoU thresholds of 0.5 and 0.75, respectively. Increasing the number of training images improves AP only decently and mainly narrows the confidence intervals. Moreover, there is only a negligible visual difference between segmentation results of the models trained on 25 and 50 images (Figures 2B–D). Training on only 10 images provides relatively good quantitative results (AP = 0.963 ± 0.021 at IoU of 0.5) but visual inspection via RedTell shows that cells are either partially segmented or completely missed (Figure 2D). Apparently, 10 images are not enough to cover the full range of cell types present in our dataset. Our experiments show that at least 25-50 images are needed to observe cells of every type during training. For datasets of larger blood smear microscopy images containing hundreds of RBCs, a training set of 25 images can be considered as a good starting point.

**FIGURE 3:**
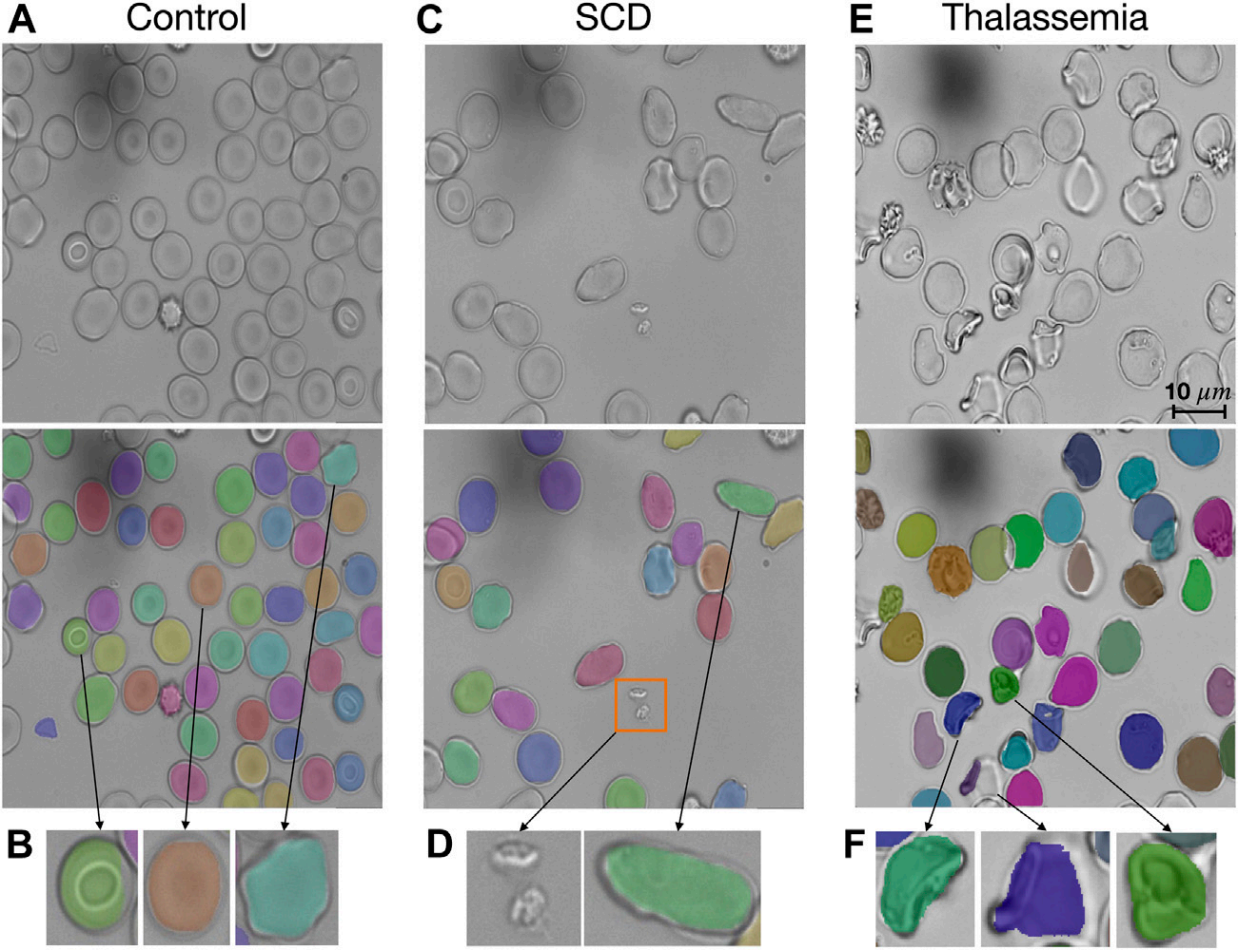
RedTell generalizes to patient images with unseen red blood cell types. **(A)** Our Mask R-CNN model was trained on red blood cell images of healthy individuals (controls) but can detect and segment different types robustly. **(B)** Stomatocytes, discocytes and echinocytes (from the left to the right) are segmented and show characteristic shapes. **(C, D)** RedTell segmentation generalizes to elongated cells appearing in SCD patients, while ignoring extra-cellular artifacts such as platelets (orange box in C). **(E, F)** The model also robustly segments cells of thalassemia patients with strongly irregular shapes.

**FIGURE 4:**
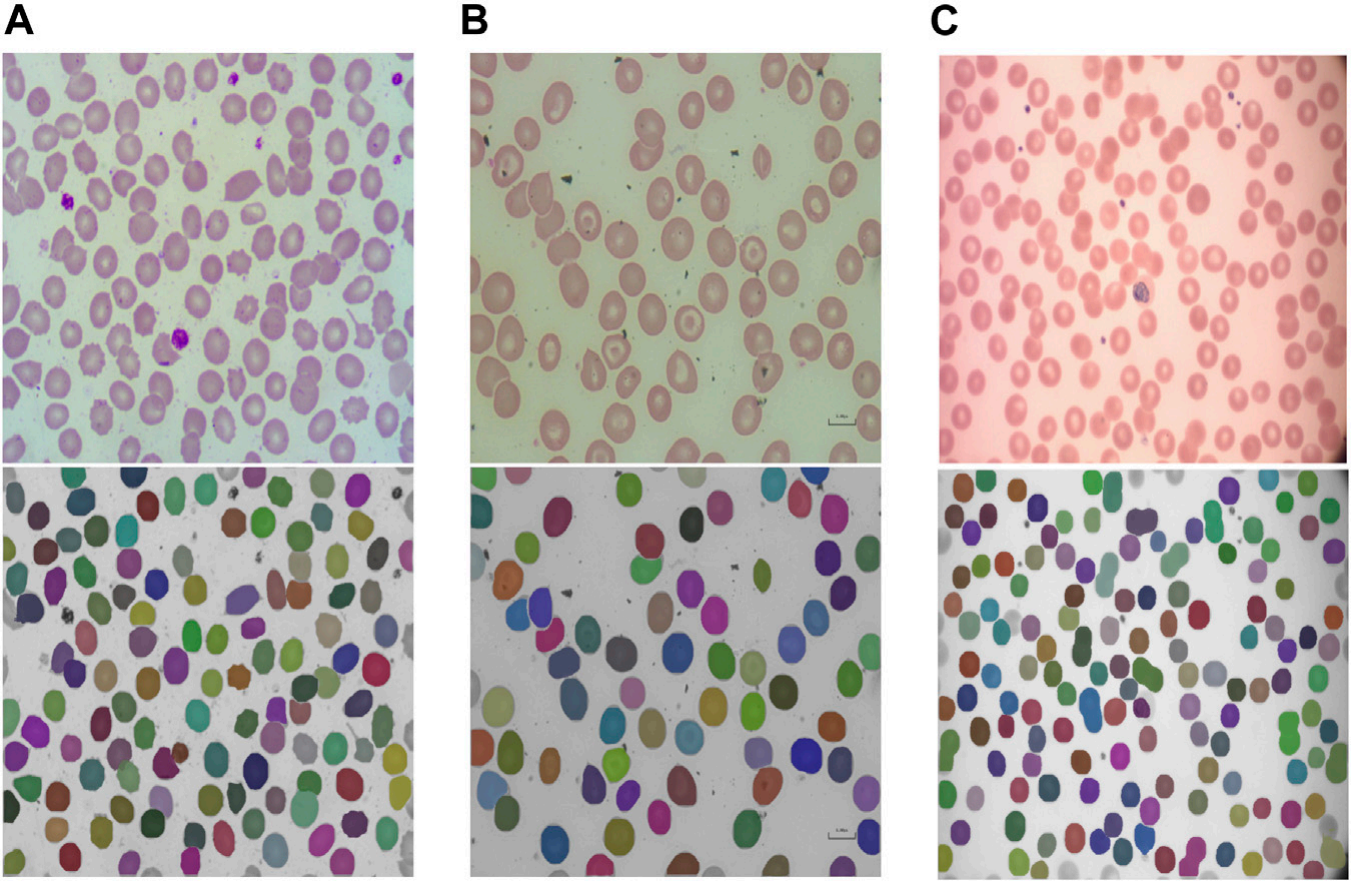
RedTell’s segmentation model trained on the CoMMiTMenT dataset accurately segments RBCs in blood smear images from three different external datasets with strong domain shifts without need for re-training. Resized raw images (top) and segmentation results (bottom) show randomly selected images from the ThalassemiaPBS **(A)**, Chula-RBC-12-Dataset **(B)**, and Malaria parasite MP-IDB **(C)** dataset.

The segmentation model provided in RedTell was trained on all 187 annotated images of the control group. We assess its generalizability in two settings: 1) internally on images of sickle cell disease (SCD) from the MemSID trial and thalassemia patients from the CoMMiTMenT dataset acquired with the same microscopy and sample preparation setup as images used for training, and 2) externally on images of three different publicly available blood smear datasets with different sample preparation and acquisition techniques as introduced in Materials and Methods. Since no ground truth annotations are available, we can only investigate the model generalizability qualitatively. Figure 3 shows the results for internal assessment, where our Mask R-CNN model properly segments irregular cells, sickle cells in case of SCD, and destroyed cells in case of thalassemia without seeing such cells in the training set. In Figure 4, we showcase segmentation results on the external datasets. Our model accurately segments cells of different shapes and overlapping cells in the images of the ThalassemiaPBS and the Chula-RBC-12-Dataset datasets. For the MP-IDB dataset, it provides accurate segmentation masks for isolated cells, but does not separate rarely presented overlapping cells.

#### Vesicle detection

**FIGURE 5:**
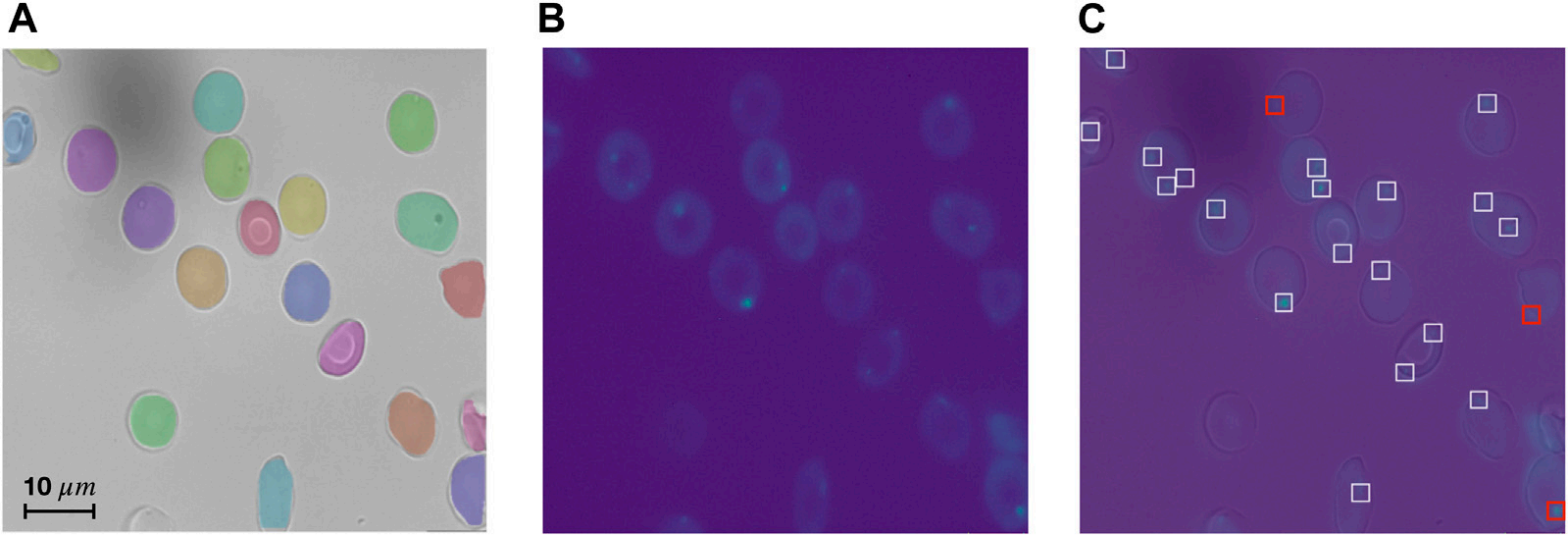
RedTell accurately detects vesicles in Ca2+ channel. **(A, B)** Test image in brightfield channel with overlaid predicted segmentation masks **(A)**, and in Ca2+ channel **(B)**. **(C)** Overlaid brightfield and Ca2+ channel with visualized vesicle detection. Most vesicles are detected correctly (white boxes), with only 3 detections being false negatives (red boxes) and no false positives.

Vesicles are remains of cell organelles due to accelerated erythropoiesis and are indicative of anemia (Alaarg et al., 2013). The number of vesicles found thus can be considered as a meaningful single-cell feature. As discussed in Materials and methods, hyperparameters of the vesicle detection algorithm need to be set for every dataset. This process is straightforward without requiring much expertise or programming. For this case study, we annotated vesicles in 6 sample images randomly selected from the CoMMiTMenT dataset and the MemSID trial, 2 samples from each class (i.e., control, SCD and thalassemia). We use 3 images to determine the optimal parameters for the algorithm with respect to the accuracy, and the remaining 3 images serve for evaluation to assess the performance of the vesicle detection algorithm. We estimate the parameters manually by calculating detection accuracy and visualizing detection results. A vesicle is considered to be correctly detected if the predicted point has an Euclidean distance of ≤5 pixels to the annotated point. Our algorithm detects vesicles in the test set with high recall (sensitivity) of 0.89 and high precision of 0.96. We provide vesicle detection results on a test image of a SCD patient in Figure 5.

### Case study 1: extracted features differ significantly between diseases

**FIGURE 6:**
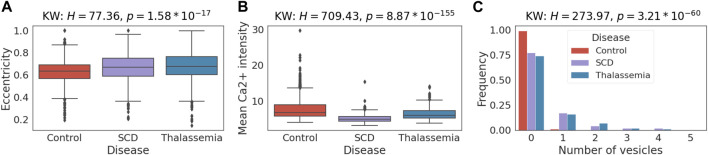
Case study 1—Single red blood cell features differ significantly between healthy and disease samples. Distribution of selected extracted features between SCD, thalassemia patients and healthy controls. H-statistics and p -values for the Kruskal-Wallis test are provided in the titles of the boxplots. The *post hoc* Dunn Bonferroni test shows significant difference (*p* < 0.05) between control-SCD and control-thalassemia pairs for eccentricity **(A)** and between all pairs for mean Ca2+ intensity **(B)** and number of vesicles **(C)**.

Here, we investigate the reasonability of extracted features and whether they align with known RBC properties or provide new insights. For simplicity, we consider only shape features, mean Ca2+ intensity, and the number of vesicles extracted from 39 images of controls, 25 images of SCD and 16 images of thalassemia patients (Figure 3) from the CoMMiTMenT and MemSID datasets. We compare the distributions of extracted features for disease groups and find that they are discriminative on the image level and can potentially be used for disease diagnosis. A Kruskal-Wallis test (Kruskal and Wallis 1952) shows a significant difference (*p* < 10^−6^) between disease groups for all selected features except the solidity ( H=7.16,p=0.03 ). For instance eccentricity, a measure of RBC cytoskeletal integrity and hemoglobin S aggregation (for SCD), of cells coming from SCD and thalassemia samples is on average higher for control group (Figure 6A). Higher values for the diseases can be explained by aggregation of Hemoglobin S known as sickling for SCD patients (Christoph et al., 2005) and by the instability of hemoglobins and premature clearance of RBCs in patients with thalassemia. Also, there is a significant difference in mean Ca2+ intensity between all three disease groups (Figure 6B) that could be explained by the hyperactivation of the plasma membrane Ca2+ ATPase resulting from its cleavage by Ca2+-dependent protease mu-calpain (Au, 1987). Moreover, in agreement with the previous observations (Bookchin et al., 1988) RBCs of SCD and thalassemia patients have more vesicles inside them to protect the cells from damage caused by enhanced Ca2+ leak through the plasma membrane (Figure 6C).

### Case study 2: discriminating cell types in control samples

**FIGURE 7:**
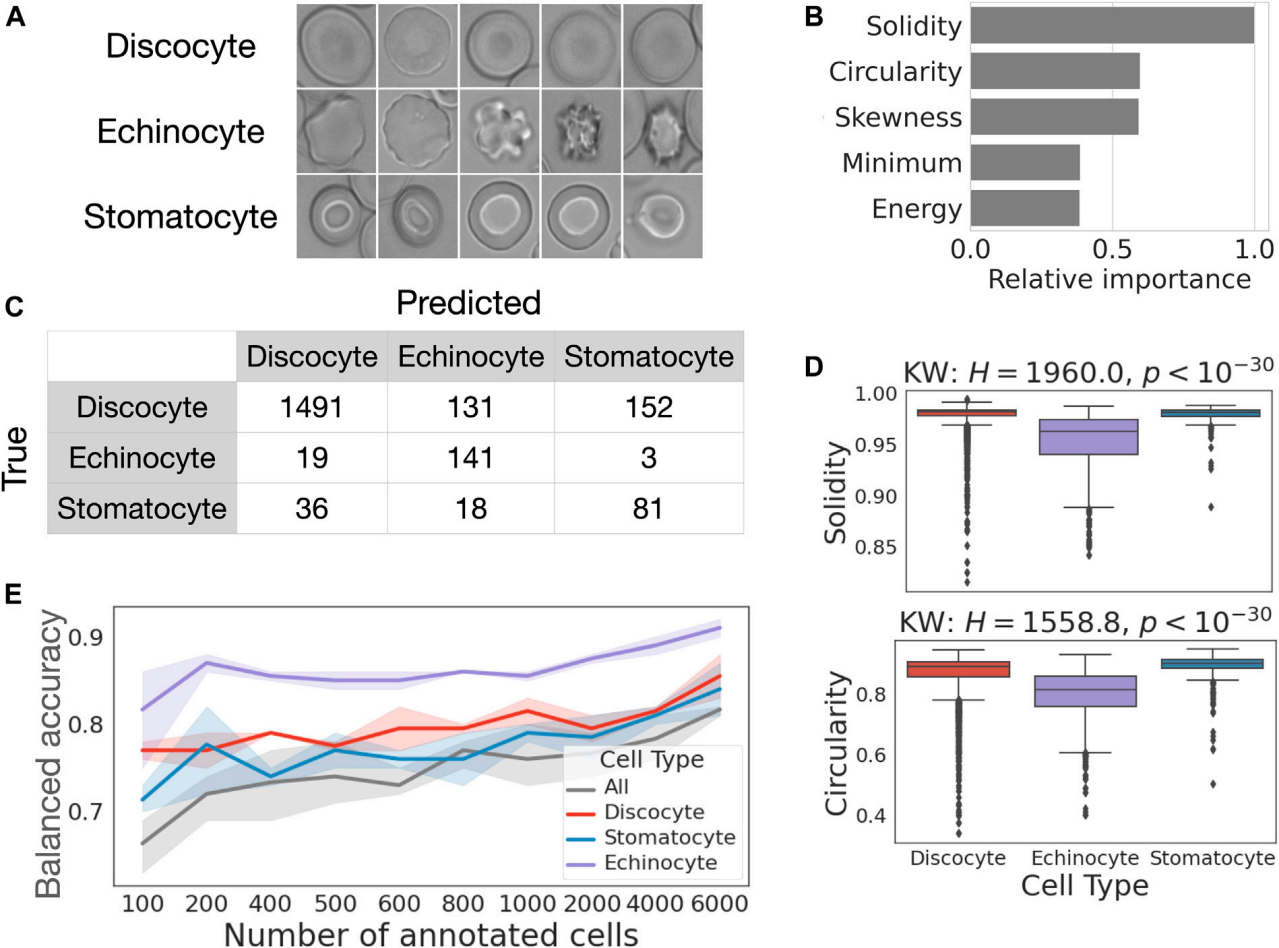
Case study 2—RedTell discriminates red blood cell types with explainable classifiers. **(A)** Example images of cell subtypes. **(B)** 200 annotated cells suffice to reach >70% accuracy for supervised cell type classification. **(C)** Confusion matrix obtained for LightGBM model, the best classifier for multi-class classification given 2000 annotated cells. **(D)** Solidity is the most important feature for the LightGBM classifier. **(E)** Solidity and circularity is significantly reduced in echinocytes.

We apply RedTell to classify stomatocyte, discocyte and echinocyte RBC subtypes (Figure 7A) in the samples from healthy controls. This case study also investigates how the number of annotated cells affects classification accuracy. The same CoMMiTMenT subset used in the segmentation experiments is annotated for classification. We first run RedTell to segment cells, to extract features and to compare detection labels with their corresponding groundtruth. We use bipartite graph matching (Bernardin and Stiefelhagen, 2008) to map centroids of the detected cells to the centroids of the ground truth annotations and match cells and determine their subtype label. We arrive at an unbalanced dataset, consisting of 8063 annotated cells, with 5% stomatocytes, 75% discocytes, and 20% echinocytes. We consider this a challenging problem for imbalanced multi-class classification as well as binary classification (one-versus-all for all three subtypes). We perform a random stratified imagewise 3 fold cross validation split into training (6000 cells) and validation (2063 cells) sets. Moreover, for every fold RedTell considers randomly stratified image-grouped sampled different fractions of training data to find the best model and evaluate on the test test. Figure 7B shows balanced accuracy achieved with the best classifiers from the RedTell Auto-ML module (decision tree, random forest or LightGBM) calculated on the test with confidence interval of 0.95 for multi-class and binary tasks. In both multi-class and binary settings, classification accuracy increases with the size of the training set. 200 annotated cells suffice to reach an average accuracy >70% for multi-class and >78% for binary classification. The highest classification accuracy of 0.92 is achieved for the echinocyte classification with 6000 cells in the training set. Increasing the size of the training set from 200 to 6000 images boosts multi-class accuracy from 72% to 82%, with an average improvement of 10%. For binary classification, this leads to an increase in accuracy between 3% and 5%. The difference between classification performance when training with 200 and 1000 annotated cells is less significant and falls within confidence intervals. Figure 7C shows the confusion matrix for the LightGBM model provided by RedTell’s Auto-ML module as the best classification model when training in multi-class settings with 4000 annotated cells. The model achieves 83% and 78% accuracy and balanced accuracy, respectively. Stomatocyte-discocyte-echinocyte fractions are given by 11%-75%-14% against true 6%-86%-8% in the test set, preserving the right order. Important features for the LightGBM classification model are provided in Figure 7D, ranked according to their relative importance and normalized by the value of the most important feature. Solidity and circularity from the shape feature set are most important, followed by three intensity-based features. Figure 7E shows the distribution of solidity and circularity across different cell subtypes. Both features are biologically reasonable and critical to differentiate echinocytes from discocytes and stomatocytes, as echinocytes are porous and have noticeably different shapes. Kruskal-Wallis and *post hoc* Dunn Bonferroni tests show significant difference insolidity between echinocytes and discocytes and between echinocytes and stomatocytes, and significant difference in circularity between all 3 cell subtypes. To differentiate stomatocytes from other cell subtypes the model relies on intensity-based features such as minimum intensity, skewness and energy.

### Case study 3: sickle cell classification

**FIGURE 8:**
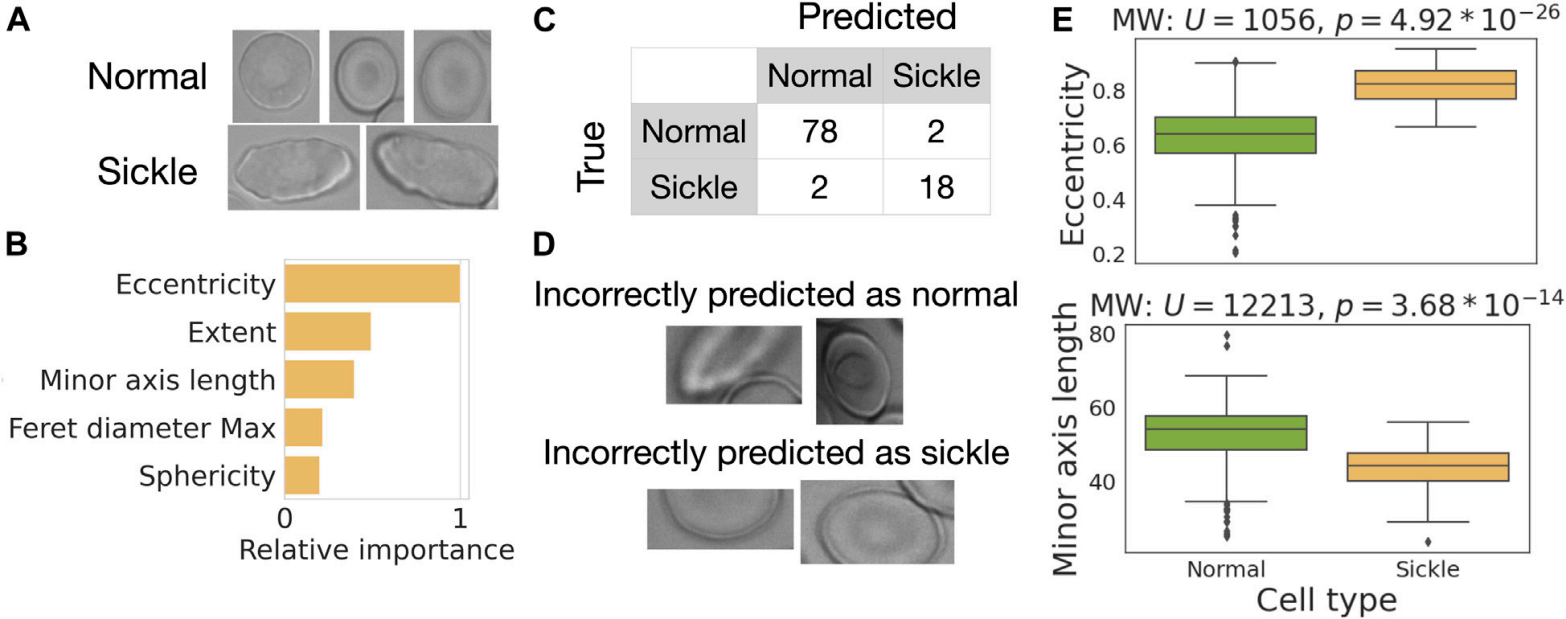
Case study—RedTell accurately detects sickle cells. **(A)** Examples of sickle and normal cells. **(B)** RedTell considers biologically meaningful features while performing classification. **(C)** Confusion matrix with only 4 cells being misclassified. **(D)** Misclassified cells are only partially visible. **(E)** Eccentricity and minor axis length show a significant difference between normal and sickle cells.

In our final case study, we use RedTell for sickle cell classification. Sickle cells have elongated forms (Figure 8A) first reported by (Herrick, 1910) and are used to diagnose SCD since then. We pick 25 images of sickle cell patients from the MemSID dataset and use RedTell to segment the cells, extract features, and randomly sample 300 cells for labeling. The class distribution in the resulting dataset is unbalanced, containing 80% normal cells and 20% sickle cells. Following the learning curve obtained for the binary classification problem in case study 2, we randomly select 200 annotated cells for training and 100 cells for testing. We use the RedTell’s Auto-ML module to train a classifier and obtain a random forest model, achieving balanced accuracy of 0.94, accuracy of 0.96 and both precision and recall of 0.90. Only 4 out of 100 cells are incorrectly classified (Figures 8C, D). One cell incorrectly predicted as normal starts to become sickle and is not elongated much, another one is on the border of the images and only partially visible Figure 8D. Cells incorrectly predicted as sickle cells are also not visible in their entirety being in the dark spots or out of focus. Partial cell visibility affects extracted features. In particular, it drastically affects the values of the classifier’s important features (Figure 8B). Random forest model uses biologically meaningful characteristics of cell morphology to differentiate between 2 cell types. The mean ranks of the two most important features, eccentricity and minor axis length, differ significantly ( $p=4.92*10^{-26}$ ; $p=3.68*10^{-14}$ Mann-Whitney-U-Test (Mann and Whitney, 1947)) for normal and sickle cells (Figure 8E).

## Discussion

We introduced RedTell, a package for automated analysis of microscopic images of RBCs. Its functionality includes RBC detection and segmentation, extraction of cell morphological properties and single cell classification. RedTell aims to accelerate research in hematology and can support diagnosis of disorders related to RBC morphologies. We showed the applicability of RedTell for three different case studies. We successfully applied it in three different case studies. First, we showed that extracted features have different distributions for healthy and anemia patients indicating feature usefulness for clinical decision making. Second, we developed a classifier to differentiate between different RBC subtypes. Finally, we used RedTell to classify sickle cells in SCD patient samples.

RedTell enables completely automated feature extraction. As for classification, it requires only expert annotation of a relatively small number of cells and no user interaction for training of the classifier. One of the main advantages of the tool is its interpretability. RedTell is interpretable tree-fold: 1) the results of cell segmentation in the brightfield channel, vesicle detection in the fluorescent channel and classification results are explicitly provided overlayed on the original images, 2) the extracted features are hand-crafted and reflect important RBCs properties, well understood and widely used by hematologists, 3) cell classification models trained within RedTell incorporate interpretability through ranking the features by their importance. Moreover, RedTell is the first RBC related work which suggests feature extraction from images of the fluorescent channels and introduces an algorithm for vesicle detection for a special case of Fluo-4 channel. In the following we will discuss single steps of the RedTell pipeline and suggest future work.

*Segmentation.* Mask R-CNN trained on the CoMMiTMenT dataset provides good results on the previously unseen images of three external datasets. We could not find any publicly available dataset, where our segmentation model would fail. However, to further increase its generalization ability to various datasets it would be reasonable to extend training of the Mask R-CNN with domain transfer methods (Zhang et al., 2022). Moreover, the segmentation part of RedTell can be extended by supporting other advanced machine learning methods, e.g., StarDist (Schmidt et al., 2018), providing a user a choice between different models to find an optimal one for a custom dataset.

*Feature extraction.* RedTell includes extraction of most of the common features widely used for the RBC analysis. Obviously, the feature set can be further extended with new features relevant to some specific user requirement, e.g., GLCM features calculated with different pixel distances and angles (Petrović et al., 2020), integral-geometry-based features (Gual-Arnau et al., 2015) or cell representation features provided by the Mask R-CNN model. Although such features are of limited interpretability, they can still improve classification accuracy.

*Classification.* We decided not to use Mask R-CNN for classification due to two reasons: 1) it requires a large amount of annotated data for a good performance, that is, necessarily labeled by an expert whose time is scarce and expensive, and 2) its performance is highly dependent on the domain and would work only with images generated under the same microscopic settings (Salehi, 2022). Although, we show that Mask R-CNN trained on our data qualitatively provides good segmentation results on the images from three external datasets (Figure 4), the same model would fail in the classification task since RBCs have different degrees of morphological details. We therefore include a classification module which takes the extracted features as input and thus allows building classification models for various datasets. Moreover, we decided to use decision tree classifiers as they resulted in high classification accuracy in previous work on RBC classification (Petrović et al., 2020) and are interpretable by design, ranking the features by their importance and hence allowing researchers to check the algorithmic logic. RedTell does not provide any classifier, but supports automated training of dataset and task specific classification algorithms. Due to numerous causes it is infeasible and beyond our goals to introduce a cell classifier, which works properly on all datasets. One important reason is interest in discrimination of different cell types (e.g., echinocyte *versus* discocyte or sickle *versus* normally shaped RBCs) depending on the available data and research question. Another limitation is varying morphology of the same cell type in microscopy images obtained under different acquisition settings. A good approach is development of a classifier specific to a given research question. Annotation of cell labels is a requirement for this task and RedTell facilitates annotation procedure. The tool includes tree-based classification algorithms which are interpretable by design. It is also possible to extend automated machine learning in RedTell with feature selection functionality to improve classification accuracy and include further classification algorithms, e.g., support vector machine (Devi et al., 2018) or deep learning-based method such as TabNet (Wong et al., 2021). However, such algorithms require advanced methods for interpretability (Altmann et al., 2010). Interpretability of the RedTell can further be improved by enabling local prediction explanation through SHAP (Lundberg and Lee, 2017) or LIME (Ribeiro et al., 2016), which provide information on what features and how they affect every single prediction.

The feature extraction and classification modules of RedTell can be applied to a broad variety of RBC research questions. In the future, we will apply RedTell to high throughput analyses, e.g., evaluating drug efficacy and assessing SCD progression by detecting and extracting features of sickle cells in blood samples of different patients at different timepoints, and hope that other researchers will follow. We also expect to include a graphical user interface for easier interaction with the user and extend RedTell functionality by providing automated analysis reports and data visualizations for the most common use cases as defined in consultation with RBC researchers.

## Data Availability

The original contributions presented in the study are included in the article/supplementary material, further inquiries can be directed to the corresponding author. We provide the datasets used for the software development, testing and case studies on Zenodo scientific sharing platform https://zenodo.org/record/7801430#.ZC1Ibi0Rr5k. The external datasets as described in the subsection “External datasets“ are publicly available.
